# How Do You Feel when You Can't Feel Your Body? Interoception, Functional Connectivity and Emotional Processing in Depersonalization-Derealization Disorder

**DOI:** 10.1371/journal.pone.0098769

**Published:** 2014-06-26

**Authors:** Lucas Sedeño, Blas Couto, Margherita Melloni, Andrés Canales-Johnson, Adrián Yoris, Sandra Baez, Sol Esteves, Marcela Velásquez, Pablo Barttfeld, Mariano Sigman, Rafael Kichic, Dante Chialvo, Facundo Manes, Tristan A. Bekinschtein, Agustin Ibanez

**Affiliations:** 1 Laboratory of Experimental Psychology and Neuroscience (LPEN), INECO (Institute of Cognitive Neurology) and Institute of Neuroscience, Favaloro University, Buenos Aires, Argentina; 2 UDP-INECO Foundation Core on Neuroscience (UIFCoN), Diego Portales University, Santiago, Chile; 3 Medical Research Council Cognition and Brain Sciences Unit, Cambridge, United Kingdom; 4 Physics Department, Laboratory of Integrative Neuroscience, FCEyN UBA and IFIBA, Buenos Aires, Argentina; 5 Anxiety Clinic, INECO (Institute of Cognitive Neurology), Buenos Aires, Argentina; 6 National Scientific and Technical Research Council (CONICET), Buenos Aires, Argentina; 7 Cognitive Neuroimaging Unit, Institut National de la Santé et de la Recherche Médicale (INSERM), Paris, France; 8 Universidad Torcuato Di Tella, Buenos Aires, Argentina; 9 Departamento de Matemáticas y Ciencias, Universidad de San Andrés, Buenos Aires, Argentina; 10 David Geffen School of Medicine, University of California Los Angeles, Los Angeles, California, United States of America; 11 Universidad Autónoma del Caribe, Barranquilla, Colombia; 12 Australian Research Council (ACR) Centre of Excellence in Cognition and its Disorders, Macquarie University, Sydney, New South Wales, Australia; Bellvitge Biomedical Research Institute-IDIBELL, Spain

## Abstract

Depersonalization-Derealization Disorder (DD) typically manifests as a disruption of body self-awareness. Interoception −defined as the cognitive processing of body signals− has been extensively considered as a key processing for body self-awareness. In consequence, the purpose of this study was to investigate whether there are systematic differences in interoception between a patient with DD and controls that might explain the disembodiment symptoms suffered in this disease. To assess interoception, we utilized a heartbeat detection task and measures of functional connectivity derived from fMRI networks in interoceptive/exteroceptivo/mind-wandering states. Additionally, we evaluated empathic abilities to test the association between interoception and emotional experience. The results showed patient's impaired performance in the heartbeat detection task when compared to controls. Furthermore, regarding functional connectivity, we found a lower global brain connectivity of the patient relative to controls only in the interoceptive state. He also presented a particular pattern of impairments in affective empathy. To our knowledge, this is the first experimental research that assesses the relationship between interoception and DD combining behavioral and neurobiological measures. Our results suggest that altered neural mechanisms and cognitive processes regarding body signaling might be engaged in DD phenomenology. Moreover, our study contributes experimental data to the comprehension of brain-body interactions and the emergence of self-awareness and emotional feelings.

## Introduction

Between 0.95% [1] and 2.4% [2] of the general population suffers from Depersonalization-Derealization Disorder (DD) [3], a syndrome characterized by a profound disruption of self-awareness [4]. Four main experiential components are described in this disorder: (1) feelings of disembodiment, which refers to the sense of detachment or disconnection from the body; (2) subjective emotional numbing, an inability to experience emotions and empathy; (3) anomalous subjective recall, a lack of ownership when remembering personal information or imagining things; and (4) derealization, an experience of feeling estranged or alienated from surroundings [4]. The description of a DD patient reflects how severe these symptoms may be: “I feel as though I'm not alive, as though my body is an empty, lifeless shell […] I seem to be walking in a world I recognize but don't feel [5].” Compared to hallucinating and delusional experiences, DD patients retain insight that these are subjective phenomena rather than the objective reality [6], [7].

Regarding the emotional and social cognition profile, DD patients rate unpleasant pictures as less emotional [8] or less arousing [9]. Based on a the Empathy Quotient (EQ) [10], a self-reported empathy scale, studies report an overall deficit in empathic abilities [11] in this disease, driven mostly by patients' lower scores in the spontaneous use of social skills and lack of intuitive social understanding [10], [11]. In the same vein, DD patients present a lack of congruent physiological arousal in response to emotive narratives [11], suggesting difficulties in *parallel* affective empathy (experience an emotion that is congruent to that of a protagonist) [12]. Research using functional magnetic resonance imaging (fMRI) reports decreased activity within neural regions engaged in emotional processing, such as the anterior insular cortex (AIC), amygdala, hippocampus, superior temporal gyrus, and anterior cingulate cortex (ACC) in DD patients while processing emotionally salient stimuli [8], [13]–[15]. Together, these studies converge to indicate that DD patients suffer from deficits in their empathic abilities and that they are unable to imbue perceived objects or concrete situations with emotional feelings [16].

In contrast to this lack of subjective emotional feelings, DD patients present an overall adequate emotional expression [4]. This discrepancy between subjective experience and the expression of emotions supports the idea that in DD there is a disruption of the process that allows emotions to gain conscious representation (usually called emotional awareness) instead of a global dysfunction of emotional processing [4].

A complementary research program has consistently established a relationship among interoception −defined as the perception of afferent visceral information from the body−, empathy and emotional awareness [17]–[27].

Subjects with higher interoceptive sensitivity rate positive and negative emotional stimuli as more arousing [22], intense [28] and stressful [25] than subjects with lower interoceptive sensitivity. Moreover, interoception seems to be related to the experienced emotion as reported in the context of everyday life [29].

Consistently, neuroimaging research shows an extensive overlap among the neural substrates underlying interoceptive, emotional and empathic experiences [17], [19], [26], [30]–[38], suggesting shared mechanisms for these processes. Brain areas most commonly involved in this network are the insular cortex (IC), the anterior cingulated cortex (ACC) and the somatosensory cortex [19], [31], [39]–[42]. The posterior and middle IC are important for mapping visceral states and the AIC integrates this visceral state with central cognitive processing [41], [43], allowing the physiological condition of the body to gain conscious representation in the form of subjective feelings [17]–[19], [43]. Somatonsesory cortex has also been described as a complementary interoceptive pathway [39], [40], and several studies support its role in pain empathy processing [44]–[46], cognitive empathy [47]–[50] emotion perception and recognition [51], [52], and understanding other's bodily state [53], [54].

Evidence of a possible relationship between DD symptoms and interoception comes from fMRI studies in normal subjects showing that the right posterior insula underpins the subjective experience of body-ownership [55], and that the feeling of losing movement control is associated with a decreased activation of this region and an increased activation of the somatosensory cortex [56]. Moreover, the somatosensory cortex has also been related to the maintaining of an on-line representation of the body [55]. Lesions' studies support the involvement of right posterior insula in the sense of limb ownership and self-awareness of actions [57], [58]. As mentioned above, the posterior insula and the somatosensory cortex are considered nodal pathways of the visceral afferents. Furthermore, these findings endorse the relationship between interoception and the representation of the body state. In consequence, if symptoms of disembodiment –similar to the ones experimented by DD patients– are associated with impairments of interoceptive awareness, it is possible that DD patients also present deficits mapping body visceral information, which might lead to an inadequate representation of their own body state. Moreover, the role of interoception in DD garners further support if we consider the presence of emotional symptoms in this disorder and the established link among interoception, emotional awareness and empathy.

Although some authors have suggested that deficits in interoception might in part underlie the symptomatology of DD [59], [60], to our knowledge, no experimental study has assessed this ability in DD patients utilizing both behavioral and neurobiological measures (fMRI connectivity analysis). Furthermore, this is the first study to assess visceral perception alongside with empathy processing in DD based on the stated relationship between both cognitive processes. Combining the two groups of literature described, we hypothesized that DD symptoms may be related to an impairment or altered system of interoception and that the physiopathology of the syndrome might be associated with deficits in the patients' perception and integration of their own visceral information, leading to an inadequate representation of their body state and, in consequence, to alterations in the emotional and empathic experience.

To examine this hypothesis, we performed an interoceptive assessment in JM, a patient with the primary diagnosis of DD. His severe anomalous body experiences and somatosensory distortions (described in Materials and Methods) represented an excellent opportunity to examine interoceptive awareness. In the study, we included cognitive tests, empathy tasks, heartbeat tracking and measures of functional connectivity derived from fMRI networks in states of interoception compared to other attentional states.

The relevance of the present study lies in its evidence of interoceptive deficits in a patient with DD utilizing both behavioral and physiological measures. Additionally, we present experimental data of the patient's impaired empathy performance. These results can contribute to the understanding of the neural mechanisms and cognitive processes underpinning DD in the context of the comprehension of brain-body interactions and the emergence of self-awareness and emotional feelings.

## Materials and Methods

### Ethics Statement

All participants signed an informed consent form before the evaluation. The patient in this manuscript has given written informed consent (as outlined in PLOS consent form) to publish these case details. The studies were conducted in accordance with the Declaration of Helsinki and approved by the INECO's ethics committee.

### Participants

#### Patient description

Patient JM is a 23-year-old male with a primary diagnosis of DD. The diagnosis was established by an expert in DD following the criteria of the revised fifth edition of the Diagnostic and Statistical Manual of Mental Disorders [3]. Additionally, JM scored over the established cut-off score (71) for the Cambridge Depersonalization Scale (CDS). Co-morbidity with anxiety disorders was assessed by means of the Structured Clinical Interview for DSM-IV axis I disorders [61]. Consistently with clinical description of DD [62]–[65], the patient met criteria for Social Anxiety and Generalized Anxiety Disorder.

His main complaints were his unremitting DD symptoms, particularly those labeled as anomalous body experiences [66]. Additionally, his voice sounded distant and unfamiliar to him and the experiential component of agency was lacking. [4].

He also presented somatosensory distortions, symptoms which are common in Depersonalization-Derealization Disorder though they are not restricted to DD. Sometimes he felt his hands were changing their size, getting either larger or smaller, and that his body was floating or levitating. These experiences invariably triggered a sense of losing control followed by distraction strategies to lessen these symptoms (e.g., listening to music).

#### Control Sample

Two groups of controls were assessed. Five healthy male controls that were matched for age and education were recruited for the neuropsychological and clinical evaluations, interoception assessment and resting fMRI scanning (interoception assessment control, IAC). A second group of five healthy male controls who were matched for age and education was evaluated with a self-reported questionnaire of interpersonal reactivity and an empathy experimental task (empathy assessment controls, EAC).

Participants from both groups did not present a history of drug abuse, neither of neurological or psychiatric conditions.

### Assessment

#### Neuropsychological and clinical evaluation

Participants from the IAC sample completed the Spanish version of the Cambridge Depersonalization Scale (CDS) [67], with the aim of eliminating any subject who obtained scores near the cutoff of this screening scale. Furthermore, we administered Beck's Depression Inventory [68] and the State Trait Anxiety Inventory (STAI) [69] to evaluate mood and affective state, respectively. Finally, this control group was evaluated with the INECO Frontal Screening (IFS) [70]. The IFS assesses executive functions as an index of the following subtasks: Motor Programming, Conflicting Instructions, Verbal Inhibitory Control, Abstraction, Backwards Digit Span, Spatial Working Memory, and Go/No Go.

#### Interoceptive behavioral measure: Heartbeat Detection Task (HBD)

We performed a motor tracking interoception test, the Heartbeat Detection Task (HBD), which has already been validated and applied in previous studies of our group [40], [71]. In the HBD participants are required to tap a computer keyboard along with their heartbeat in different conditions. This motor tracking task was selected based on its differences, and advantages for our research, when compared to traditional interoceptive sensitivity paradigms. The heartbeat discrimination task [72] involves a possible interference factor [73] introduced by the nature of the instructions that request participants to attend both their endogenous heartbeat sensations and auditory or visual cues. Regarding the other traditional interoceptive paradigm, mental tracking [24], the HBD has the advantage of measuring correct and incorrect answers and to evaluate participants performance after an auditory feedback.

During the HBD the ECG signal was recorded with an ad-hoc circuit composed of an amplifier AD620 and a band-pass filter (low 0.05 Hz, high 40 Hz) and then analogically fed to a laptop computer's audio card. Three Ag/Ag-Cl adhesive electrodes were placed to every subject in lead-II positions together with headphones for audio stimuli delivery. The signal was processed on-line with a PsychToolbox [74] script, running on Matlab platform (MathWorks). External electrodes were used in the ECG setup to collect the ECG signal, which was processed in real time for peak detection and audio stimulation following the heartbeats.

This experimental task began with the assessment of two control conditions of motor response skills. In the first condition, participants were instructed to follow an audio recording of a sampled heartbeat that presented a constant frequency of beats (60 bpm). In the second one, they had to follow a recorded audio that was previously manipulated to have a variable and inconstant frequency. Next, they were told to follow their heartbeat two times with no external stimulation or feedback (first and second interoceptive condition). Then, they were given the same instructions along with simultaneous auditory feedback of their heart provided through online ECG register (feedback condition). Finally, they were once again told to follow their heartbeat without any feedback, and this instruction was also repeated twice (third and fourth interoceptive condition).

Using a measure of accuracy response, we compared participants' performance across the conditions to determine whether they were following or not their heartbeats sensations (see Data processing and analysis below).

#### Body mass-index

Previous studies reported that interoception performance may depend on the body mass index (BMI) [75]. To control the possible biases of this bodily difference, we measured the BMI in all participants.

### Interoceptive fMRI scanning: acquisition

Functional images were acquired on a Phillips Intera 1.5T with a conventional head coil. Thirty-three axial slices (5 mm thick) were acquired parallel to the plane connecting the anterior and posterior commissures and covering the whole brain

(TR = 2777 ms, TE = 35 ms, flip angle = 90).

JM and the IAC sample were scanned under three resting state conditions that lasted ten minutes each: exteroception, mind wandering and interoception. The instructions of the first condition requested participants to focus on the sequence of sounds generated by the noise of the scanner and to silently count them. The goal of this instruction was to manipulate their attention to focus it directly on the exogenous stimulus. In the next condition, the mind wandering or proper resting state condition, subjects were told to think about what they had done that day since waking or what they were going to do for the rest of the day. Finally, in the interoceptive condition participants were instructed to focus on their respiration cycle and on their heartbeats. In all three conditions, subjects were told to keep their eyes closed and to avoid moving and falling asleep.

### Empathy Tasks

#### Self-report questionnaire: Interpersonal Reactivity Index (IRI)

The patient and subjects from the EAC group completed the interpersonal reactivity index (IRI) [76], a 28-item self-reported questionnaire that measures both the cognitive and affective components of empathy. This scale comprises four subscales: 1) Fantasy (F), assesses the extent to which participants identify themselves with fictional characters; 2) Perspective Taking (PT), evaluates the extent to which individuals try to adopt another's point of view; Empathic Concern (EC), measures the feelings of warmth, compassion and concern for others; Personal Distress (PD), assesses the feelings of anxiety and discomfort when faced with a negative experience from another individual.

#### Empathy for pain (EPT)

This task evaluates empathy in the context of intentional and accidental harm [40], [77]–[80]. In this test, 24 animated situations are shown to the participants (see Video S1). Each situation depicts one of three kinds of interactions between two people: a situation where one person intentionally hurts (active performer) another person (passive performer), e.g., someone hits a person with a bat on the stomach on purpose (intentional pain situation); another kind of situation where a person hurts another one by accident (accidental pain situation), e.g., a person goes backwards with his bike and accidentally hurts someone else; and a third type of interaction where two people interact in a neutral connotation situation (control situation), e.g. one person gives a book to another one [80].

Following the video, the participants are asked to press a button as soon as they have understood the situation and then they are asked to answer seven questions: (1) Was the action done on purpose? [evaluating cognitive aspects of empathy (intentionality); answered selecting Yes/No]; (2) How sad do you feel for the hurt person? [evaluating affective aspects of empathy (empathic concern)]; (3) How upset do you feel for what happened in the situation? (evaluating discomfort towards the situation); (4) How bad person the perpetrator is? [evaluating the intention of the perpetrator to hurt the victim (harmful behavior)]; (5) How happy do you feel for the person that committed the action? (evaluating the valence towards the behavior); (6) How inappropriate was the action? (evaluating correctness of the action) and (7) How much penalty would you impose on the perpetrator? (evaluating the moral aspects of empathy and punishment). Questions two to seven were answered using a computer–based visual analogue scale (VAS) that rates from -9 to 9 (see Video S1). The meaning of the scale extremes depends on the question, for example on the question “how sad do you feel for the hurt person?” one extreme of the bar reads “I feel very sad” and the other extreme reads “I don't feel sad at all”. Accuracy and RT were measured for the first question, and ratings (*empathy-related judgments*) and RT for questions two to seven were measured. The RT measured the time that passed from the moment the question appeared, to the time the participant answered. There was no pre-determined interstimulus interval as each stimulus would start as soon as the participants had answered the last question of the previous item. Before testing, all participants performed a trial session with a similar situation in order to ensure the correct understanding of the instructions.

### Data processing and analysis

#### Heartbeat Detection Task (HBD)

To assess performance on the HBD, we used an index that normalizes the subjects correct responses based on the total amount of heartbeats (Accuracy Index). This index allows us to compare participants without the bias of heart rate differences. We utilized a modified equation from the one proposed by Schandry for his heartbeat mental tracking method [24]. Schandry employs the total amount of mental heartbeats counted and the total number of heartbeats recorded as measures for his index.

Our motor tracking method allows us to discriminate the correct motor response of the participants from their incorrect answers. To separate them, every motor response was compared within a specific time window around every recorded heartbeat; if the tapping input was temporally located within the corresponding time window for each beat, the response was considered as correct (the time window is determined by the subjects' heart rate: between 0.125 milliseconds before and 0.750 milliseconds after the beat, for a heart frequency less than 69.76; between 0.1 milliseconds before and 0.6 after, for frequencies between 69.75 and 94.25; and 0.075 milliseconds before and 0.4 milliseconds after, for frequencies higher than 94.25). The total sum of all of the subjects' responses that fulfilled this temporal criterion was considered as correct answers, and we used this more specific measure of interoceptive performance instead of the total sum of responses.

Thus, the accuracy equation we used is:
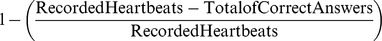



We calculated an Accuracy Index for every condition of the task that can vary between 0 and 1 with high scores indicating smaller differences between correct answers and recorder heartbeats.

### FMRI preprocessing and graph theory analysis

#### Preprocessing

Functional data were preprocessed using statistical parametric mapping software (SPM8; http://fil.ion.ucl.ac.uk/spm). EPI images from all sessions were slice-time corrected and aligned to the first volume of the first session of scanning to correct head movement between scans. Movement parameters showed no movements greater than 3 mm or rotation movements higher than 3 degrees of rotation [81]. T1-weighted structural images were first co-registered to a mean image created using the realigned volumes. Normalization parameters between the co-registered T1 and the standard MNI T1 template were then calculated, and applied to the anatomy and all EPI volumes. Data were then smoothed using a 8 mm full-width-at-half-maximum isotropic Gaussian kernel to accommodate for inter-subject differences in anatomy (these proceedings were followed according to the pre-processing steps described in another paper of our group: [82]).

#### Correlation matrices

First, based on a 116-Atlas [83], mean time courses were extracted by averaging BOLD signal of all the voxels contained in each of the 116 regions of interest (ROI). These averages fMRI time series were then utilized to construct a 116-node functional connectivity (FC) network for each subject and condition. Wavelet analysis was used to construct correlation matrices from the time series [84]. We followed the same procedures described by Supekar et al. [84] and employed in other work from our group [82]. First, we applied a maximum overlap discrete wavelet transform (MODWT) to each of the time series to establish the contributing signal in the following three frequency components: scale 1 (0.13 to 0.25 Hz), scale 2 (0.06 to 0.12 Hz), and scale 3 (0.01 to 0.05 Hz). Scale 3 frequencies lie in the range of slow frequency correlations of the default network [85], [86], thus connectivity matrices based on this frequency were utilized for all posterior analyses. Each ROI of these connectivity matrices corresponds to a node, and the weights of the links between ROIs were determined by the wavelets' correlation at low frequency from scale 3. These connectivity matrices describe time frequency-dependent correlations, a measure of functional connectivity between spatially distinct brain regions.

#### Graph theory metrics: Global Networks

To calculate network measures from FC, we applied the same procedure used in previously published works [82], [87]–[89]. This methodology involves converting the weighted functional matrices into binary undirected ones by applying a threshold T on the correlation value to determine the cutoff at which two ROIs are connected. We used a broad range of threshold correlation values from 0.0005<T<1, with increments of 0.001. The outputs of this procedure were 1000 binary undirected networks for each one of the three resting macro-states (exteroception, resting and interoception). Then, the following network measures were calculated using the BCT toolbox [90] for each binary undirected matrices: a) degree (k), represents the number of connections that link one node to the rest of the network [91]; b) the characteristic path length (L), is the average of the minimum number of edges that must be crossed to go from one node to any other node on the network and is taken as a measure of functional integration [92]; c) average clustering coefficient (C) indicates how strongly a network is locally interconnected and is considered a measure of segregation [92] and d) small-world (SW) that refers to an ubiquitous present topological network which has a relatively short (compared to random networks) characteristic path length (L) and high average clustering coefficient (C) [92].

Instead of using the small-world (SW) measure from this toolbox, which was proposed by Humphries and Gurney [93] and involves the calculation of random networks, we combined integration (L) and segregation (C) metrics into a single formula to calculate the *small-worldness* of the network [82]:
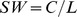



This decision is based on Rubinov and Sporns' [94] suggestion that Humphries and Gurney's SW measure may falsely report a small-world topology in very poorly integrated networks. Thus, to avoid biases from networks with these topological characteristics, they recommend considering the individual assessment of integration and segregation when characterizing functional connectivity matrices, as we did with the SW formula employed.

For the statistical analysis of the 1000 binarized networks per subject, we only used the range between the 50^th^ network to the 800^th^ (excluding the extreme values where network disaggregate) and created 15 steps or bins based only in their metric values. Each bin or step consisted in a given range comprising fifty binarized matrices (e.g., setp or bin one 51–100; step two 101–150, etc.) in which we calculated an average of all metrics measures. The results of these procedures were 15 averaged metrics values ((800–50)/50)) per subject and per condition.

#### Graph theory metrics-Interoceptive-emotional Network

To specifically compare brain areas related to interoceptive and empathy processing, we analyzed the local metrics of three regions of interest (ROIs): IC, ACC and somatonsensory cortex. Therefore, instead of using all the 116 areas comprised in the Tzourio-Mazoyer anatomical atlas [83], we selected these three anatomical areas bilaterally. Based on the same procedure described above, we selected metrics that bring information about the segregation of each ROI: a) local clustering coefficient (lC), that quantifies the number of existing links between the nearest neighbors of a node as a proportion of the maximum number of possible links [92], and b) the local efficiency (E), defined as the inverse shortest path length within the nearest neighbors of the node in question [95]. We ran the same statistical analysis procedure used for the global metrics analysis but for these two metrics.

#### Network size

Creating binary and undirected matrices by applying a threshold to determine the correlation cutoff of connections among ROIs involves the generation of networks of different sizes. For example, a particular threshold could determine that a group of ROIs is connected in one weight matrix and not in another. Accordingly, when these two matrices are binarized using this threshold, they will present a different amount of ROIs connected among each other. Different functional network sizes using this method depend on the ROIs' correlation strengths for each individual subjects, and this might bias the network characterization when graph metrics are calculated. To control this bias, we also applied another process to generate binary and undirected matrices. Instead of establishing a particular threshold for brain correlations, we used the number of links (ROIs connected) in the weighted network as a cut-off to create each undirected graph. We utilized a broad range of connection values ranging from networks with one connection up to networks that were fully connected, with increments of 6728 connections to create 1000 undirected graphs. As we did in the previous processes for the statistical analysis, we used a broad range of connection values, from 50 to 800 connections, in steps of 50 (excluding the extreme values where networks disaggregate).

All our data analysis (neuropsychological and clinical evaluations, interoceptive behavioral measure, fMRI resting-state images and empathy for pain results) are available upon request.

### Statistical analysis

To compare the patients' performances with both control samples, we used a modified one-tailed t-test [96]–[99]. This methodology allows the assessment of significance by comparing multiple individual's test scores with norms derived from small samples. Although parametric statistics usually requires comparison groups of about 30 subjects, the one-tailed t-test allows the assessment of significance by comparing an individual's score to the scores obtained in a small control sample (even less than 5 subjects) [99]. This modified test is more robust for non-normal distributions, presents low values of type I error, and has already been reported in recent single case studies [40], [100], [101]. Additionally, this statistical method has already been employed in an early investigation that compared the connectivity indices of fMRI during resting states between a small control sample and a single case [102].

### Procedure

Patient JM was first evaluated via a psychiatric examination by an expert on Depersonalization-Derealization disorder and anxiety disorders (R.K). Next, JM and each participant from the IAC sample were assessed with the HBD task during individual sessions. All of the evaluations took place in a noise-free and comfortable environment. Additionally, in the same session, we administered the neuropsychological test (IFS) and the self-report questionnaires (BDI, STAI and CDS). In another session, JM and participants from this group underwent fMRI scanning. In the second step of the study, the patient and the second control group, EAC, were evaluated using empathy tasks (IRI and EPT) in individual sessions.

## Results

### Sociodemographic, clinical and neuropsychological results

Sociodemographic, clinical and neuropsychological results of JM and the IAC sample are provided in Table 1. No significant differences in age (t = −1.52, p = 0.1, Z_cc_ = −1.67), years of formal education (t = −0.76, p = 0.24, Z_cc_ = −0.84) and gender (they were all males) were found between JM and the IAC group. No patient-control differences were observed in either the neuropsychological EF evaluation (IFS) (t = −1.56, p = 0.09, Z_cc_ = −1.70), depression (t = 0.91, p = 0.21, Z_cc_ = 0.99) and anxiety state and trait (STAI-S, t = 1.26, p = 0.14, Z_cc_ = 1.38; STAI-T, t = 0.87, p = 0.21, Z_cc_ = 0.96).

**Table 1:** Demographic, clinical and neuropsychological assessment.

	JM		T	p	Zcc	IAC Simple
*Sociodemographic data*						
**Age**		23	−1.52	0.1	−1.67	M = 28.2; SD = 3.11 (25–33)
**Formal education (in years)**		16	−0.76	0.24	−0.84	M = 17.4; SD = 1.67 (15–19)
IFS						
**Total Store**		23/30	−1.56	0.09	−1.70	M = 27; SD = 2.34 (25–30)
*Affective screening*						
**Depression (BDI)**		8	0.91	0.21	0.99	M = 2.8; SD = 5.21 (0–12)
**Anxiety State (STAI-S)**		28	1.26	0.14	1.38	M = 26.2; SD = 1.30 (25–28)
**Anxiety Trait (STAI-T)**		39	0.87	0.21	0.96	M = 30.2; SD = 9.20 (22–46)

### Cambridge Depersonalization Scale

**Figure 1:**
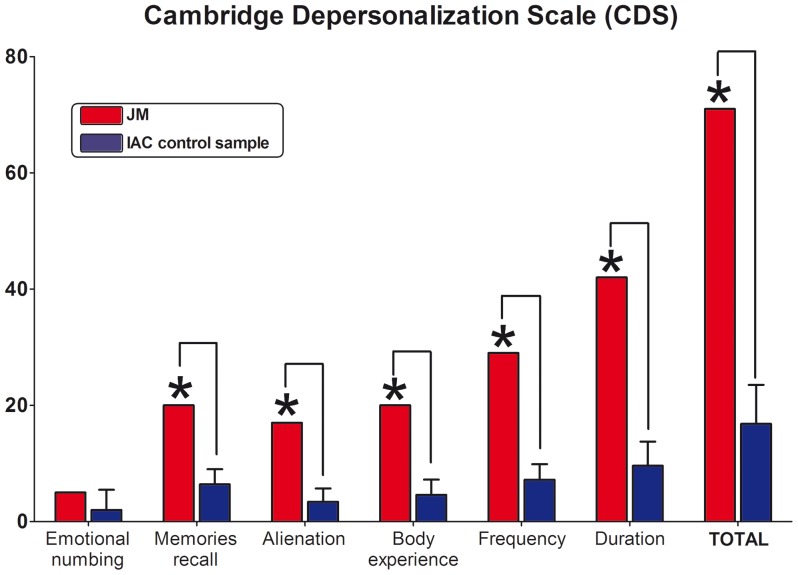
Cambridge Depersonalization Scale (CDS). Subscales and Total Raw Scores. Higher scores in the first four subscales represent a higher presence of experiences from each of the DD main symptoms (all significant, except for Emotional Numbing). Frequency and duration refer to all DD symptoms. Total score is a product of the sum of the measures, and its established score cut off is 70. * expressed significant differences between DD patient and control sample.

JM showed significant differences from the IAC group in almost all of the subscales of the CDS that measure the intensity of the subjective experience of depersonalization symptoms (memories recall, t = 4.76, p<0.01, Z_cc_ = 5.21; alienation, t = 5.40, p<0.01, Z_cc_ = 5.91; body experience, t = 5.39, p<0.01, Z_cc_ = 5.92), except for emotional numbing (t = 0.79, p = 0.24, Z_cc_ = 0.87). Additionally, JM presented significantly higher scores compared to controls in the subscales of the CDS that assess frequency (t = 7.41, p<0.01, Z_cc_ = 8.13) and duration (t = 7.11, p<0.01, Z_cc_ = 7.78) of depersonalization-derealization episodes. Finally, significant differences were found between the patient and controls in the total score (t = 7.36, p<0.01, Z_cc_ = 8.06) (see also Fig. 1).

### Interoceptive results

#### Heartbeat Detection Task (HBD)

**Figure 2:**
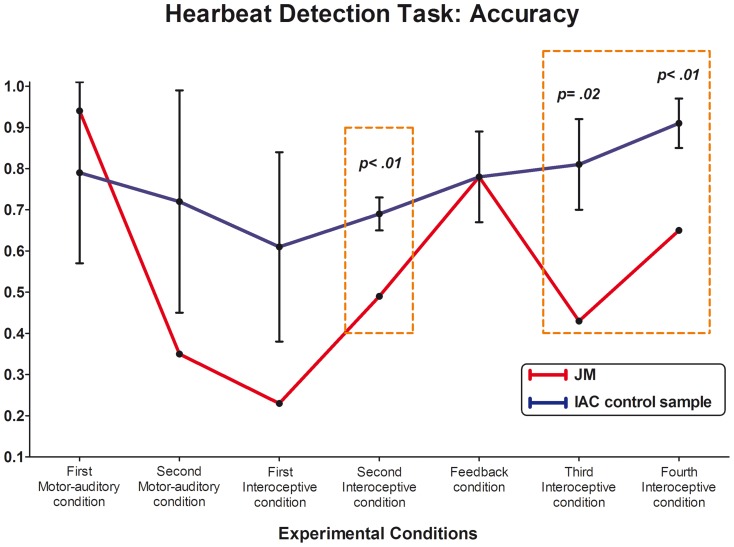
Heartbeat Detection Task (HBD). The Accuracy Index can vary between 0 and 1, with higher scores indicating better interoceptive sensitivity. * indicates significant differences between JM and the control sample.

No significant differences were found between the patient and the IAC sample in the first two motor-auditory conditions (first motor-auditory t = 0.62, p = 0.28, Zcc = 0.68; second motor-auditory t = −1.25, p = 0.14, Zcc = −1.37). In these conditions, participants were told to follow recorded heartbeats. Similar results were obtained when comparing the patient's and controls' performance in the first interoceptive condition (t = −1.50, p = 0.10, Zcc = −1.65). However, controls showed a significantly higher Accuracy Index than the patient in the second interoceptive condition (t = 0.49, p<0.01, Zcc = −5). In these conditions, participants were told to follow their own heartbeats without any auditory cue. In the following condition, where subjects listen online to their own heartbeats through headphones, both groups presented similar results (t = 0, p = 0.50, Zcc = 0). Finally, significant differences were found in the last interoceptive conditions; as in the second interoceptive condition, controls exhibited a higher Accuracy Index than the patient (third interoceptive condition, t = −3.15, p = 0.02, Zcc = −3.45; fourth interoceptive condition t = −3.96, p<0.01, Zcc = −4.33). In these, subjects were requested to concentrate on their physical sensations again and to follow their own heartbeats without any cue (see also Fig. 2).

In summary, JM exhibited a deficit performance, compared to IAC sample, in almost all interoceptive conditions, and both groups only showed similar results in conditions that involved following some auditory cue (first and second motor-auditory condition as well as feedback conditions).

#### Body Mass Index

No significant differences in body masss index (BMI) were found between the patient and this control sample (t = 0.78, p = 0.24, Zcc = 0.85).

### Interoceptive Functional Connectivity (FC) Results

The small size of the IAC group represents one possible limitation of the fMRI analysis. To test whether the five subjects of this group could be used as a representative control sample, we compared their mind-wandering FC with that from 23 normal subjects (age, gender, and handedness matched) extracted from the 1000 Functional Connectomes Project [103], an open-access repository of resting-state functional MRI datasets (http://fcon_1000.projects.nitrc.org/). The results showed no differences between the IAC sample and controls from the connectomes project, suggesting that our sample group might be representative of a more general healthy population (see Information S1 for details of these analyses and Figure S1 for results).

### Comparing network connectivity matrices

**Figure 3:**
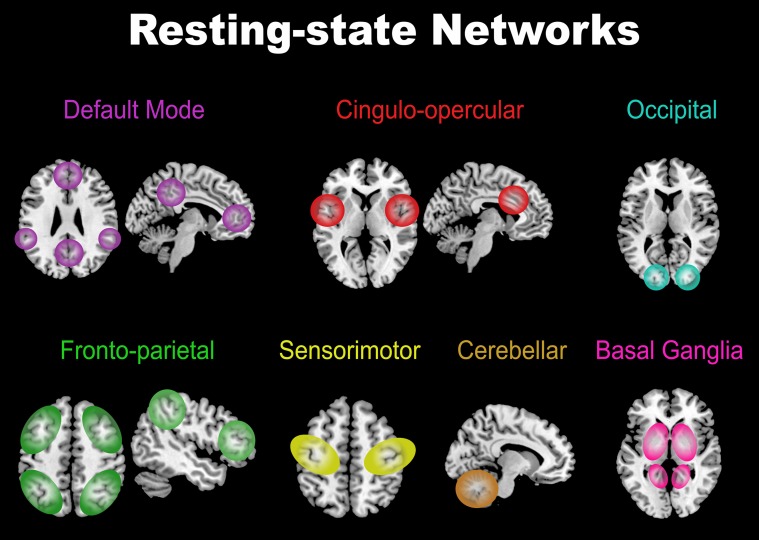
Resting-state networks. Most-often reported networks in previous research that contain groups of brain regions highly correlated with each other.

Functional connectivity matrices describe the relationship between brain regions that are anatomically separated but functionally linked during resting states. From the vast amount of spontaneous brain activity arise different networks that comprise groups of brain regions that are highly correlated with each other [104]–[106]. These networks are usually referred to as resting-state networks (see [107] for a review of this networks). Fig. 3 illustrates the most often reported resting-state networks including the default mode network (consisting of the precuneus, medial frontal and inferior parietal and temporal regions), the cingulo-opercular network (temporal/insular and anterior cingulate cortex regions), the occipital or visual network, the fronto-parietal network (superior parietal and superior frontal regions), the primary sensorimotor network, the basal ganglia and the cerebellum [108]–[114].

**Figure 4:**
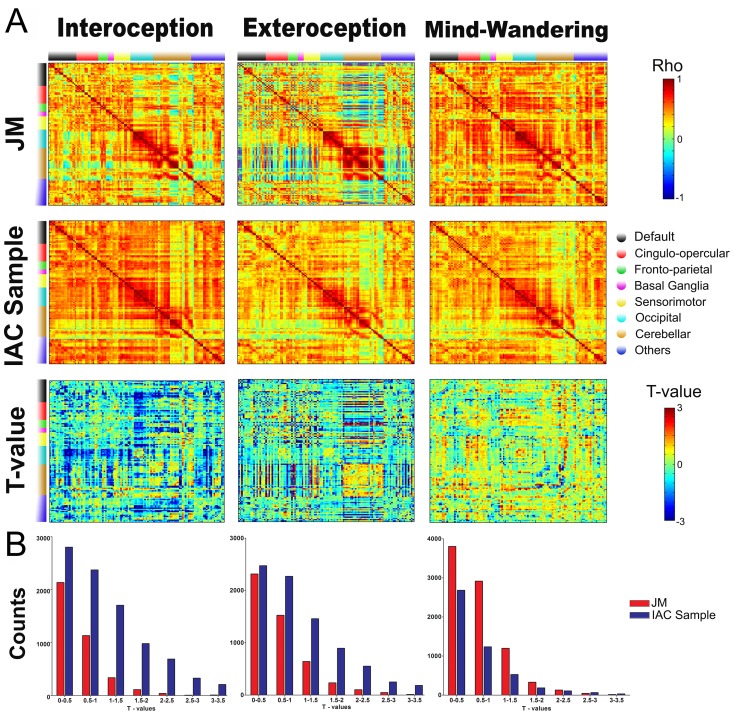
Networks connectivity matrices. (A) Averaged correlation matrices for JM, control sample and conditions. Bottom rows shows t-values for test-t between JM and the control group. (B) T-value distributions for JM (red) and the IAC sample (blue).

These standard resting-state networks are labeled in our functional brain connectivity matrices (see Fig. 4). Thus, for each connectivity matrix (exteroception, interoception and mind-wandering), we conducted a modified one-tailed t-test for each entry of the matrix comparing the patient and the IAC (see Fig. 4). A positive t-value indicates increased connectivity in the patient compared to the IAC sample. Conversely, a negative t-value indicated a greater connectivity in controls than in the patient.

The distribution of absolute t-values is shown in the Fig. 4, which visualizes an unsigned estimate of change across groups for each cognitive state. To test the connectivity between JM and controls in these distributions of t-value matrices, we performed a one-sample t-test. Our null hypothesis was that the distribution matrices came from a distribution with mean zero, which would indicate no difference in the connectivity between groups being compared across the three cognitive states. The results of this t-test rejected the null hypothesis in the three states. Negative t values found in exteroceptive (mean  = −0.48, std  = 1.38, t = −40.74, CImin  = −5.08, CImax  = −0.46) and interoceptive condition (mean  = −0.73, std = 1.37, t = −61.60, CImin  = −0.75, CImax  = −0.70) suggests that JM presented a strong decreased connectivity pattern compared to controls. Contrarily in the resting condition, positive t-values reflect an increased connectivity in JM compared to controls (mean  = 0.19, std = 0.89, t = 25.22, CImin  = 0.18, CImax  = 0.21).

These results show relevant differences in the large-scale brain functional organization across different cognitive/attentional states between JM and the control group. Despite of the fact that these outcomes are presented across the three resting-states, t-values suggest that mean connectivity differences among brain areas might be more pronounced in the interoceptive condition.

### Graph theory metrics: Global Networks

**Figure 5:**
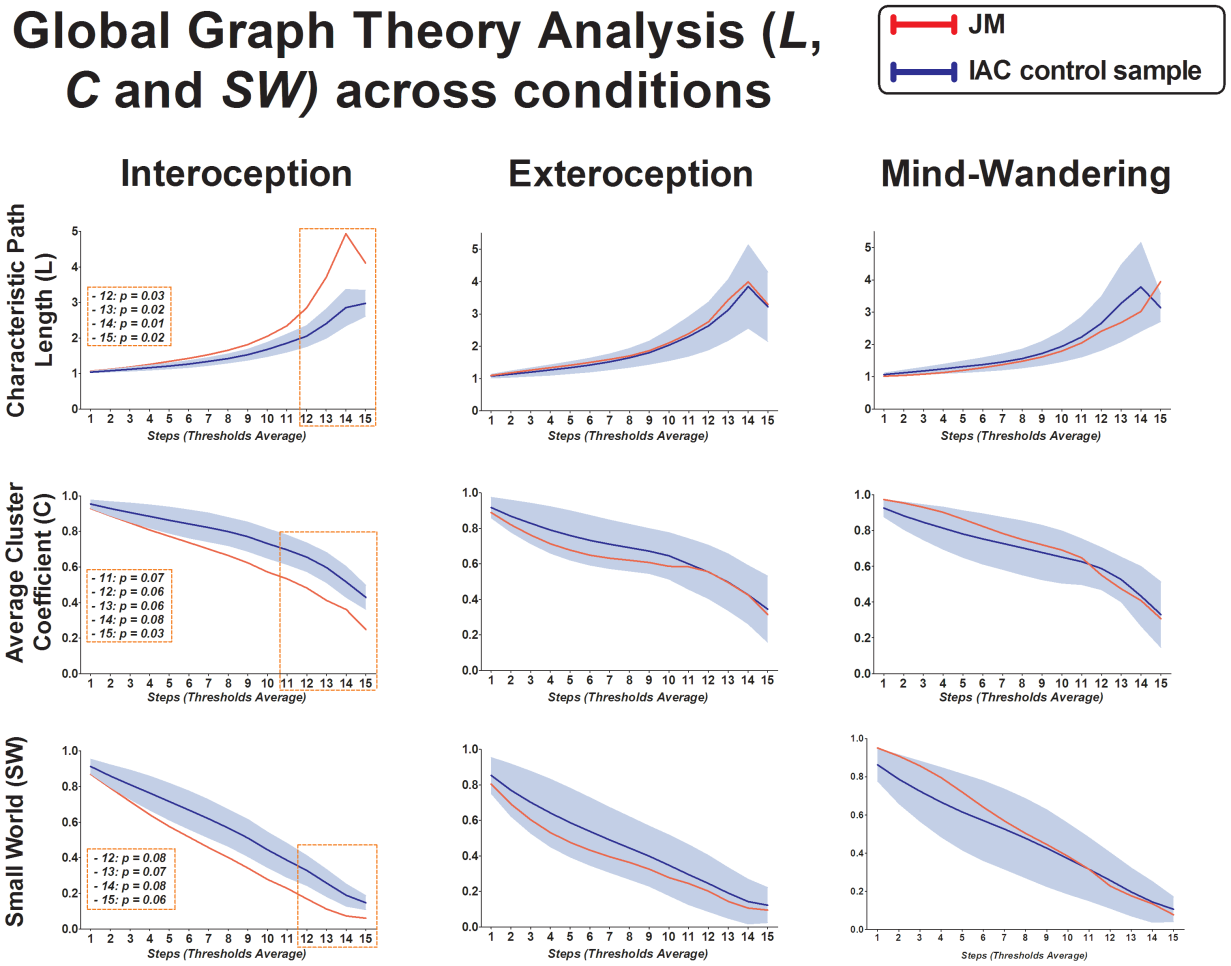
Global Graph Theory Analysis. Columns indicate each resting-state condition, and rows indicate each graph metric. The Y-axis shows raw metric scores, and the X-axis shows the range of thresholds, from 50 to 800, in steps of 50 (excluding extreme values where networks disaggregate). *Boxes indicate significant and trend differences between JM and the control sample. Blue shadows represent controls*' *standard deviation area.*

No significant differences in any network measures were found between the patient and the IAC group throughout the 15 steps in either the mind-wandering or the exteroceptive macro-states. However, a comparison between groups in the interoceptive condition revealed that JM has a higher characteristic path length (L) than controls in all of the steps (presenting significant differences in the last four: 12, t = 2.47, p = 0.03, Z_cc_ = 2.70; 13, t = 2.88, p = 0.02, Z_cc_ = 3.15; 14, t = 3.70, p = 0.01, Z_cc_ = 4.05; 15, t = 2.85, p = 0.02, Z_cc_ = 3.12). The patient also showed a decreased average clustering coefficient (C) compared to controls, although only trend differences were found in the last four steps and just one significant result in the last one (11, t = −1.81, p = 0.07, Z_cc_ = −1.98; 12, t = −1.97, p = 0.06, Z_cc_ = −2.164; 13, t = −1.99, p = 0.06, Z_cc_ = −2.19; 14, t = −1.64, p = 0.08, Z_cc_ = −1.79; 15, t = −2.46, p = 0.03, Z_cc_ = −2.70) (see Fig. 5).

Regarding the small-world (SW), no significant differences were found between JM and controls throughout the three cognitive states, however controls presented a trend toward higher SW organization in the interoception condition in the last four steps (12, t = −1.73, p = 0.08, Z_cc_ = −1.89; 13, t = −1.77, p = 0.07, Z_cc_ = −1.95; 14, t = −1.71, p = 0.08, Z_cc_ = −1.87; 15, t = −1.99, p = 0.06, Z_cc_ = −2.19) (see Fig. 5). Fig. 5 shows that this trend was only found in this cognitive state and not in the others (exteroception and resting), where the SW organization between groups was similar.

Finally, the degree (K) did not evidence differences in any of the conditions.

### Graph theory metrics: Local Networks

**Figure 6:**
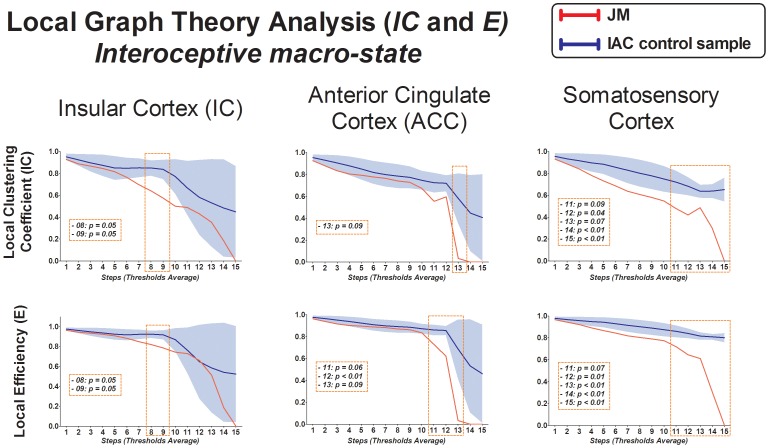
Local Graph Theory Analysis − Interoceptive macro-state. Columns indicate each ROI from the interoceptive-emotional network, and rows indicate each graph metric. The Y-axis shows raw metric scores, and the X-axis shows the range of thresholds, from 50 to 800, in steps of 50 (excluding extreme values where networks disaggregate). *Boxes indicate significant and trend differences between JM and the control sample. Blue shadows represent controls' standard deviation area.*

In this analysis we compared the local metric of ROIs from the interoceptive-emotional network previously defined: IC, ACC and somatonsesory cortex. No metrics differences were found in this network in the mind-wandering macro-state neither in the exteroceptive condition (see Figure S2 and Figure S3 for results). Regarding the interoceptive condition, the patient exhibited similar metrics results to the ones found in the graph analysis of the global network. During this last attentional macro-state, we found a decreased local clustering coefficient (lC) and local efficiency (E) in JM's network topology compared to controls. This pattern of decreased segregation metrics was presented in all the ROIs (differences were mostly in the last steps of the analysis): IC, ACC and somatonsensory cortex (see Fig. 6 and Information S2 for detailed results).

### FC controlling by the network's sizes

Given that we created a serial of networks with similar node size, no differences were found in the degree (K) of any of them in any condition. The K of a ROI represents the number of connections that link it to the rest of the network [91]. Indeed, this network measure is the criterion we utilized to create the undirected graphs during this process; this is why no differences were found.

Additionally, a similar event occurred with the characteristic path length (L) of these networks that showed no significant differences between groups. L, which is defined as the average of the minimum number of ROIs that must be traversed to go from one ROI to all the others in the network [91], is highly influenced by K values. The distance that separates ROIs depends on the number of network connections. If more areas are connected within the network, smaller is the distance to travel from one ROI to all the others. Thus, if we compared networks that present the same number of connections, then the average distance that separates one ROI from the others might be similar. In this way, this might explain why we did not find differences in L when comparing JM with controls in any of the cognitive states.

In conclusion, no differences were found neither in K or L when the size of networks was controlled. Additionally, both the patient and controls presented similar results in the remaining graph metrics (C and SW) during the exteroception and resting states. However, controls showed a significantly higher C than JM (relevant results in most of the steps: 2, t = −2.63, p = 0.03, Z_cc_ = −2.89; 3, t = −3.06, p = 0.02, Z_cc_ = −3.36; 4, t = −3.91, p<0.01, Z_cc_ = −4.30; 5, t = −2.71, p = 0.03, Z_cc_ = −2.97; 6, t = −2.55, p = 0.03, Z_cc_ = −2.81; 7, t = −2.34, p = 0.04, Z_cc_ = −2.56; 8, t = −2.12, p = 0.05, Z_cc_ = −2.32; 9, t = −2.02, p = 0.06, Z_cc_ = −2.22) and also an increased SW measure (trend differences in three steps: 9, t = −2.01, p = 0.06, Z_cc_ = −2.21; 10, t = −1.76, p = 0.08, Z_cc_ = −1.92; 11, t = −2.02, p = 0.08, Z_cc_ = −1.92; and significant differences in one: 12, t = −2.29, p = 0.04, Z_cc_ = −2.51) during the interoception condition.

To summarize, after applying the correlation threshold procedure, JM presented a significantly higher characteristic path length (L) than controls, and trended toward a lower average clustering coefficient (C) and lower Small World (SW) only during the interoceptive condition. The patient also showed a significant decreased clustering coefficient (lC) and local efficiency (E) in the analysis of the interoceptive-emotional network (IC, ACC and somatosensory cortex) during interoceptive macro-state exclusively.

Metrics results from the correlation threshold procedure are consistent with those found in networks of similar sizes (where the number of connections was used instead of correlation thresholds to control and normalize networks size). In this control procedure, JM also exhibited trends of lower C and SW exclusively during the interoceptive condition, suggesting that differences in the large brain scale organization between the patient and the IAC sample are not biased by different networks size [81].

### Empathy Tasks Results

#### Interpersonal Reactivity Index (IRI)

**Figure 7:**
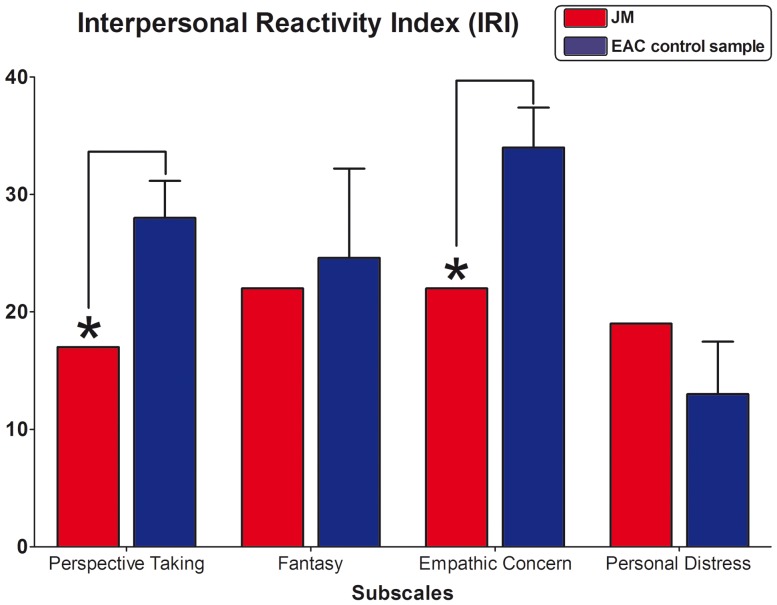
Interpersonal Reactivity Index (IRI). Subscales raw scores. * indicates significant differences between the DD patient and the control sample.

JM scored lower on the PT subscale (Perspective Taking, t = −3.17, p = 0.02, Z_cc_ = −3.48) and on the EC subscale (Empathic Concern, t = −3.23, p = 0.01, Z_cc_ = −3.45) than the EAC sample. No significant differences were found in the PD (Personal Distress, t = 1.22, p = 0.14, Z_cc_ = 1.34) and F (Fantasy, t = −0.31, p = 0.38, Z_cc_ = −0.34) subscales between groups (see also Fig. 7).

### Empathy for pain (EPT)

**Figure 8:**
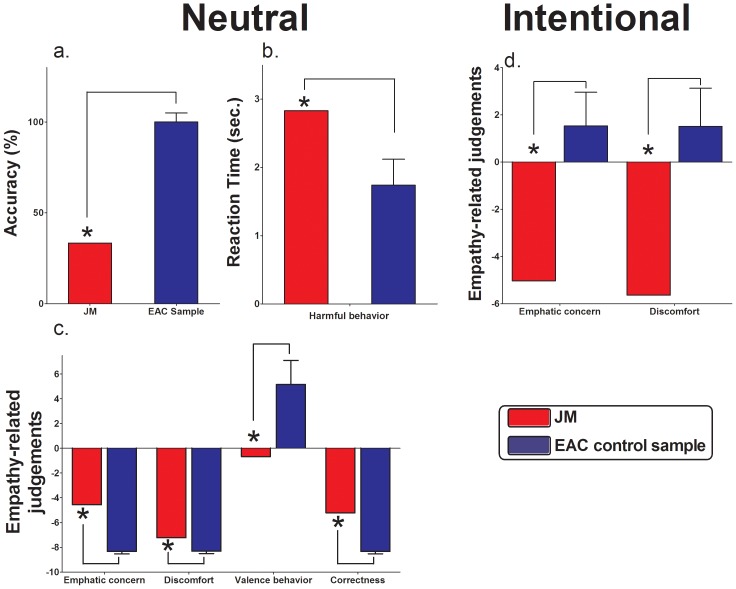
Empathy for pain task (EPT). Neutral condition results: (A) categorization accuracy in percent; (B) reaction time in seconds of Harmful behavior and (C) average pain rating scores for each question after scenes of the neutral condition. Intentional condition results: (D) average empathy-related judgments scores for each question after scenes of this condition. * expressed significant differences between the DD patient and control sample.

JM showed some patterns of impairments in EPT associated with the recognition of neutral and intentional conditions compared to the EAC sample. In the first condition, he presented deficits in the recognition of action intentionality (*t* = −60.87, *p*<0.01, *Zcc* = −66.67), significantly lower RTs in harmful behavior (*t* = 2.59; *p* = 0.03; *Zcc* = 2.84), lower empathy-related judgments in valence behavior (*t* = −2.72; *p* = 0.02; *Zcc* = −2.98) and higher empathy-related judgments in empathic concern (*t* = 3.44; *p* = 0.01; *Zcc* = 3.77), discomfort (*t* = 20.04; *p*<0.01; *Zcc* = 22.24) and correctness (*t* = 2,84; *p* = 0.02; *Zcc* = 3.11). In the second condition, he simply exhibited lower empathy-related judgments in empathic concern (*t* = −4.18; *p*<0.01; *Zcc* = −4.59) and discomfort (*t* = −4,02; *p*<0.01; *Zcc* = −4.40) (see also Fig. 8 and Information S3 for a table with detailed description of results).

## Discussion

The goal of this study was to assess interoception in a patient with chronic DD. The main finding was that the patient presented deficits on the cognitive processing of body signals both in a behavioral interoceptive task and during an fMRI interoceptive macro-state. In addition, to test the link between interoception, empathy and DD, we utilized empathic tasks, where JM showed an impaired performance based on his inadequate empathic responses to scenes depicting neutral and harmful situations.

This is the first experimental research that directly assessed the link among DD symptoms, empathy and interoception combining behavioral and neurobiological measures. The results of interoceptive deficits in JM may contribute to the understanding of cognitive processes and neural underpinnings of DD. Together with empathic results, they become a source of evidence for the comprehension of emotion-interoception interactions and for the emergence of self-awareness and emotional feelings.

### Interoception and DD

JM experienced a high intensity of symptoms regarding body disengagement, as shown by both the CDS results and by his own clinical complaints. Based on this phenomenology and on the relationship between self-awareness and interoception, we proposed the hypothesis of interoceptive deficits in JM, which was then supported by results in the HBD task and in the functional connectivity analyses.

The HBD is a measure of interoception: higher accuracy scores on this task are associated with better interoceptive sensitivity. Compared to controls, JM presented a worse performance in conditions involving the detection of one's endogenous heartbeat, without external cues. This behavioral evidence sustains our hypothesis about interoceptive sensitivity impairments in the patient.

In the same vein as the behavioral measures, functional connectivity analyses of interoceptive macro-states showed a consistent trend of lower global brain connectivity of JM compared to controls. These results were supported by the analyses of the connectivity between brain areas in each macro-state and by its characterization using graph theory metrics. In the former, network connectivity matrices showed that the highest differences between JM and control sample among resting-state conditions were presented in the interoceptive one, where the patient exhibited a less connected network compared to controls.

Despite that these matrices analysis presented differences across all cognitive states between groups, exclusively during the interoceptive state, JM's brain connectivity network revealed sub-optimal metrics: higher characteristic path length (L), lower average clustering coefficient (C) and lower small-world (SW). A SW organization is an important feature of brain network complexity that reflects an optimal balance of a high level of segregation (C) with a high level of global integration (L) [115]. Metrics results showed that DD patient exhibited lower levels of segregation (C) than controls which might imply an altered efficiency in local information transfer and processing. Additionally, the higher characteristic path length (L) of the patient might indicate impairments of network functional integration that refers to the combination of specialized information rapidly from distributed long-range connections. This disruption of global and local functional networks in the patient compared to controls suggests a loss of efficiency in information exchange between both regional and distributed brain areas and, therefore, an altered global topological organization of brain network only during interoceptive macro-state.

In order to analyze the brain connectivity within areas specifically involved in interoceptive and emotional processing, we compared metrics of segregation of the IC, ACC and the somatosensorial cortex between the patient and normal subjects. As we found in the global assess of functional networks, JM presented lower levels of segregation (local clustering coefficient, lC, and local efficiency, E) in these ROIs only during the interoceptive macro-state. These results highlight the possible impairment in local processing of interoceptive information within this network. The relevance of the disruption of these interoceptive-emotional ROIs is based on the fact that group's differences were found only when participants were requested to focus attention on their cardiac and breathing sensations, and not when they attended to external sounds or they thought about their daily routine. In consequence, these connectivity deficits in global patterns and in key interoceptive ROIs, during the attention to the endogenous stimuli of heart and breathing, might indicate an ineffective system for the integration and processing of interoceptive information.

In light of previous neuronanatomical findings, the association found in this patient between interoceptive deficits −in our behavioral and neurobiological measures− and disembodiment symptoms garners further support. Neuroimaging studies have shown that better performance in the HBD task engaged higher activation of the right AIC and the ACC [19], [23]. The right AIC area is critical for self-awareness [41], [43]: it integrates the flow of interoceptive information from the posterior and middle parts of the IC with central cognitive processing, allowing the physiological condition of the body to obtain conscious representation in the form of subjective feelings [17], [18], [30]. Consequently, worse interoception sensitivity might be associated with decreased activation of IC. Additionally, a lesion study [39] highlighted the role of the somatonsensory cortex as part of another interoceptive pathway involving skin afferents projections. A patient with complete bilateral IC and ACC damage, but intact bilateral primary somatonsesory cortex, demonstrated interoceptive awareness comparable to healthy controls. However, when a topical lidocaine anesthetic was applied to the skin covering the region of maximal heartbeat sensation, only control participants presented changes in interoceptive awareness. As a result, authors proposed the existence of two interoceptive awareness pathways: one compressing visceral afferents projections to the insula and the other involving skin afferents projections to somatosensory cortex [39].

The plausibility of this relationship between these interocceptive hubs and DD disembodiment symptoms is further suggested by recent studies that have shown that the subjective experience of body-awareness is associated with the IC and somatosensory cortex [55]–[58]. In consequence, our findings about connectivity deficits in the IC, ACC and somatonsesory cortex during the interoceptive macro state, together with their role in interoceptive and body awareness, suggest the possible involvement of this brain network as a neural substrate for DD.

In summary, behavioral and neurobiological data support our prediction of interoceptive awareness impairments in JM. This deficit would lead to alterations in the process whereby the visceral body state gains conscious representation in the form of self-awareness and emotional feelings. In this way, it may be possible that DD disembodiment symptoms are partly associated with alterations in interoceptive mechanisms. Moreover, IC, ACC and somatosensory cortex, which are engaged in interoception and self-awareness, may be considered as a neural substrate of DD [11], [59].

### Relevance for state-of-the-art models of DD and interoception

The possible role of interoception in DD can be linked with the two-network neurobiological model of DD [4]. First, an abnormal prefrontal regulation of the AIC [4] is considered to be responsible for emotional numbing symptoms. Second, based on phenomenological overlaps between symptoms of brain-injured patients and DD, it is suggested that disrupted parietal functioning would account for disembodiment in DD [116]. Furthermore, as we have already mentioned, the same neural systems are revealed as two independent pathways related to interoception: one involving an AIC-ACC network and the other implicating parietal regions (S1 and S2) [39]. The confrontation of anatomical areas involved in each of these models highlights the possible association between interoception −and its underlying brain network comprised by IC, ACC and somatosensory cortex− and DD symptoms. Additionally, an interoceptive model of conscious presence [59] directly proposed that DD symptoms might be related to imprecise body signal predictions. Our findings provide experimental evidence for this model proposal about the interoceptive deficits in DD patients.

### Empathy and DD

Although JM's main clinical complaints did not include abnormalities in his emotional experiences, and no differences were found in the CDS emotional numbing subscale, he presented impairments in the experimental assessment (EPT) of affective empathy. In first place, he failed to recognize the intentionality of neutral acts when compared to controls. This difference might be due to the fact that neutral scenes are less salient and more ambiguous than accidental and, especially, intentional ones [78]. Thus, lack of stimuli salience [26] in this condition may have represented an obstacle for the patient to elucidate the intention of actors in the scene and, consequently, could have induced his altered pattern of empathy-related judgments (see Fig. 8). On the other hand, the most interesting results of this task correspond to patient's performance during the intentional condition, where stimuli depicted people that are harmful intentionally in violent ways. When asked about his empathic −“*gut feeling*”− reactions against what happened in these scenes, he experienced significantly less empathic concern (sadness) and discomfort for victims of intentional harm. In the same line, JM reported difficulties in his capacity to feel compassion for others (IRI sub-scale: Empathic Concern, EC). These last results highlight, despite the absence of complains about emotional numbing, that the patient might present deficits in the affective component of empathy.

Embodied views of affective empathy [11], [117]–[119] state that a principal component of empathy relies on the generation, representation and perception of one's own feeling state. Evidence for the relationship between affective empathy and feeling states comes from studies showing a neural overlap between both cognitive processes, mainly involving the IC [120]–[123]. If the understanding of others' experience is to some extent related to the perception of one's own internal state [124]–[126], then disruptions in the processing of one's own feelings may have an impact on empathic response. Given this situation, DD patients with emotional numbing should present empathic impairments, as proven by DD studies that found a patients' diminished emotional reaction to other's feelings [11] and impairments in implicit measures of empathic abilities [10].

Our findings about JM's less empathic response are consistent with these reports and, to our knowledge, are the first data from an experimental design (EPT) that directly assessed the empathic response to highly affective scenes. However, the experimental results did not seem to be related to JM's complaints given that he did not express any clinical difficulties in his emotional sphere. One possible explanation for this lack of clear emotional numbing symptoms, along with the presence of experimental deficits in empathic experiences, could be related to his disembodiment symptoms. Extreme feelings of anomalous body sensations could have minimized the presence of emotional difficulties during clinical assessment.

Related to the cognitive dimension of empathy, the results from the IRI suggest that JM presented deficits in adopting others' point of view. This finding differs from the DD literature where unimpaired performances of patients have been reported in cognitive empathy [11]. One possible explanation of this divergence is that former studies utilized traditional tasks without any social context (e.g., the “reading the mind in the eyes, [127]), instead, the IRI sub-scale evaluates the skill to take the outer perspective in “real life” situations [128]. Difficulties in this task are expected, as previous findings in DD reported deficits in empathic skills within social situations [10]. Consequently, DD patients might present spared cognitive empathy when social context is not involved and deficits in tasks consisting of social situations that introduce more complex scenarios (where it is harder to disentangle the cognitive and affective components).

In sum, despite the fact that emotional numbing symptoms were not clearly presented in the clinical assessment of JM, the experimental evaluations found deficits in affective and cognitive components of empathy. Embodied views of cognition, which state the relationship between emotional feeling awareness and affective empathy, along with the interoception-emotions interaction, highlight the possible role of interoceptive deficits in empathy impairments.

### Interoception, empathy and DD

Interoceptive processing contributes to the basis of emotional experience and feeling state. Within an embodied view of empathy, interoception, as the representation of bodily internal states linked with emotional experiences, could be involved in processing the affective state of others. Recent findings sustain this prediction [21], showing the modulation of cortical processing of heartbeats by the affective judgment of facial stimuli. Furthermore, an fMRI study showed the enhancement of empathy-related activity in the bilateral IC, posterior to interoceptive awareness [129]. In the case of the somatosensory cortex, several studies indicated its role in pain empathy processing (especially when physical injuries are involved) [44]–[50], [55] and in interoception awareness [39]. Those results support the close association between interoception and empathy, and provide evidence about the impact of body signal processing in the experience and manifestation of feelings for others. In the same vein, JM's deficits in interoception and empathy are consistent with this association between the body and subjective emotional feelings, reflecting that impairments in the perception and integration of visceral information might lead to inadequate representation of feelings states and to disruptions in affective empathic response. Moreover, a recent fMRI research [60] that compared processing of facial emotional expression between DD patients and controls showed a relationship between alexithymia and brain areas related to interoception, monitoring and reflection of internal states and emotion. These findings support our experimental results about interoceptive and emotional deficits in JM and highlight the possible substantial role of interoceptive impairments in the phenomenology of DD regarding body and emotional awareness.

### Limitations and further directions

The current research presents several limitations that should be accounted for in future studies. First, inferences are based on evidence from a single case, which is not enough to completely clarify the role of interoception and empathy in DD for which future group studies shall be conducted. Moreover, the small sample size of the control group and the presence of two different control samples are issues that might bias our inferences about the cognitive mechanism underlying DD phenomenology. To clarify our proposal of interoceptive deficits as a key impaired process in DD, it is necessary to utilize larger control groups in future research. Additionally, our research only assessed empathy experiences; future studies should also use tasks that evaluate a broad range of emotional experiences to gain a deeper insight into the interaction between interoception and emotions. Finally, despite JM met criteria for Social Anxiety and Generalized Anxiety Disorder −according to the module F of the Structured Clinical Interview for DSM-IV Axis I Disorders−, the possible interference of anxiety or depression levels was controlled, and patient did not present differences in this affective domain compare to controls. Furthermore, several studies have stated that patients with DD often present high level of anxiety [62], of which more common manifestations are social anxiety and panic attacks [63]–[65]. Despite this association, a recent study suggest that the relationship between anxiety and DD is non-significant in patients with moderate to severe symptoms of DD [130].

Nevertheless, this is the first experimental report that combines the assessment of interoception and affective empathy in a DD patient with extreme disembodiment symptoms. In this way, we provide direct evidence of the association between interoception and DD phenomenology that should be further tested, representing an important step to better understand the neural mechanism and substrates of this disabling condition.

## Conclusions

DD is a syndrome characterized by a disruption of body self-awareness. Our behavioral and neurobiological evidence of impaired interoceptive awareness and affective empathy in JM support the association between these affective-cognitive domains. Alongside theoretical and empirical evidence from DD and body/self-awareness research, these results highlight the possibility that altered interoceptive processing is engaged on the basis of clinical features, such as disembodiment and emotional numbing. In consequence, body signal processing, which leads to conscious representation of the self and of emotional states, would contribute to the understanding of the cognitive mechanisms and neural substrates underlying DD phenomenology.

## Supporting Information

Figure S1 **Graph Theory Analysis of mind-wandering resting-state.** 1000 Functional Connectomes Project vs. IAC sample (FC metrics). The Y-axis show raw metrics score and the X-axis, the range of thresholds, from 50 to 800, in steps of 50.
(TIF)

Figure S2 **Local Graph Theory Analysis − Mind wandering macro-state.** Columns indicate each ROI from the interoceptive-emotional network, and rows indicate each graph metric. The Y-axis shows raw metric scores, and the X-axis shows the range of thresholds, from 50 to 800, in steps of 50 (excluding extreme values where networks disaggregate). *Boxes indicate significant and trend differences between JM and the control sample. Blue shadows represent controls' standard deviation area.*
(TIF)

Figure S3 **Local Graph Theory Analysis − Exteroceptive macro-state.** Columns indicate each ROI from the interoceptive-emotional network, and rows indicate each graph metric. The Y-axis shows raw metric scores, and the X-axis shows the range of thresholds, from 50 to 800, in steps of 50 (excluding extreme values where networks disaggregate). *Boxes indicate significant and trend differences between JM and the control sample. Blue shadows represent controls' standard deviation area.*
(TIF)

Information S1 **1000 Functional Connectomes Project's analysis and results.** Description of the analysis and results of the comparison between the control sample and the data from the 1000 Functional Connectome Project.
(DOC)

Information S2 **Graph theory metrics: Local Networks of Interoceptive Condition.** Detailed results of graph analysis of the IC, ACC and somatonsesory cortex during the interoceptive condition.
(DOC)

Information S3 **Empathy for Pain (EPT) Results.** Table with the complete results of this task.
(DOC)

Video S1 **Examples of the Stimuli of the Empathy for pain (EPT).** Examples of intentional, accidental and neutral conditions of the task.
(WMV)
