# Supplementary figures and images for: How Do You Feel when You Can't Feel Your Body? Interoception, Functional Connectivity and Emotional Processing in Depersonalization-Derealization Disorder
Source: PLoS One. 2014 Jun 26;9(6):e98769. doi: 10.1371/journal.pone.0098769 (PMC4072534; doi:10.1371/journal.pone.0098769)

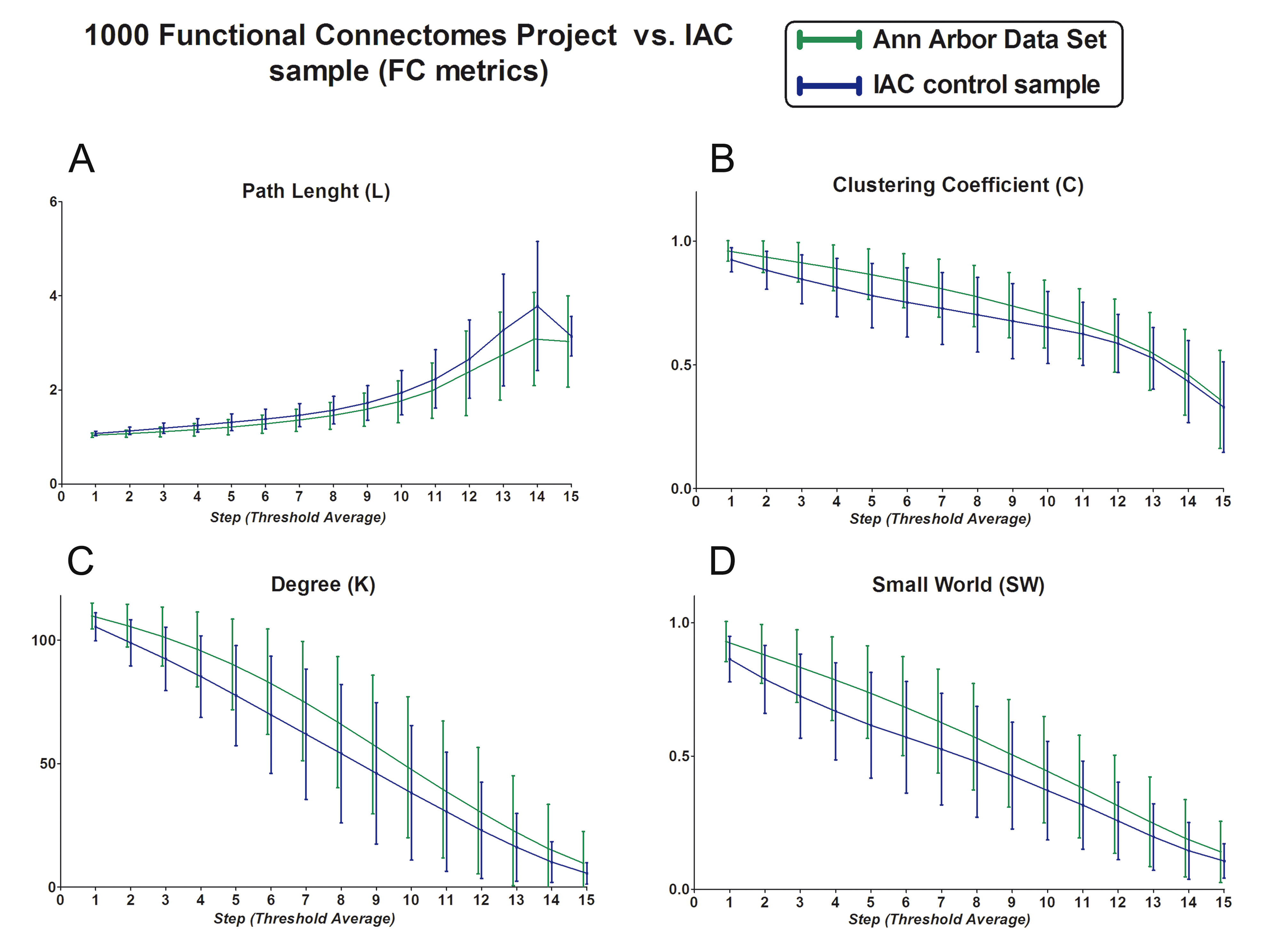

Supplement: Figure S1 — Graph Theory Analysis of mind-wandering resting-state. 1000 Functional Connectomes Project vs. IAC sample (FC metrics). The Y-axis show raw metrics score and the X-axis, the range of thresholds, from 50 to 800, in steps of 50. (TIF) [file pone.0098769.s001.tif]

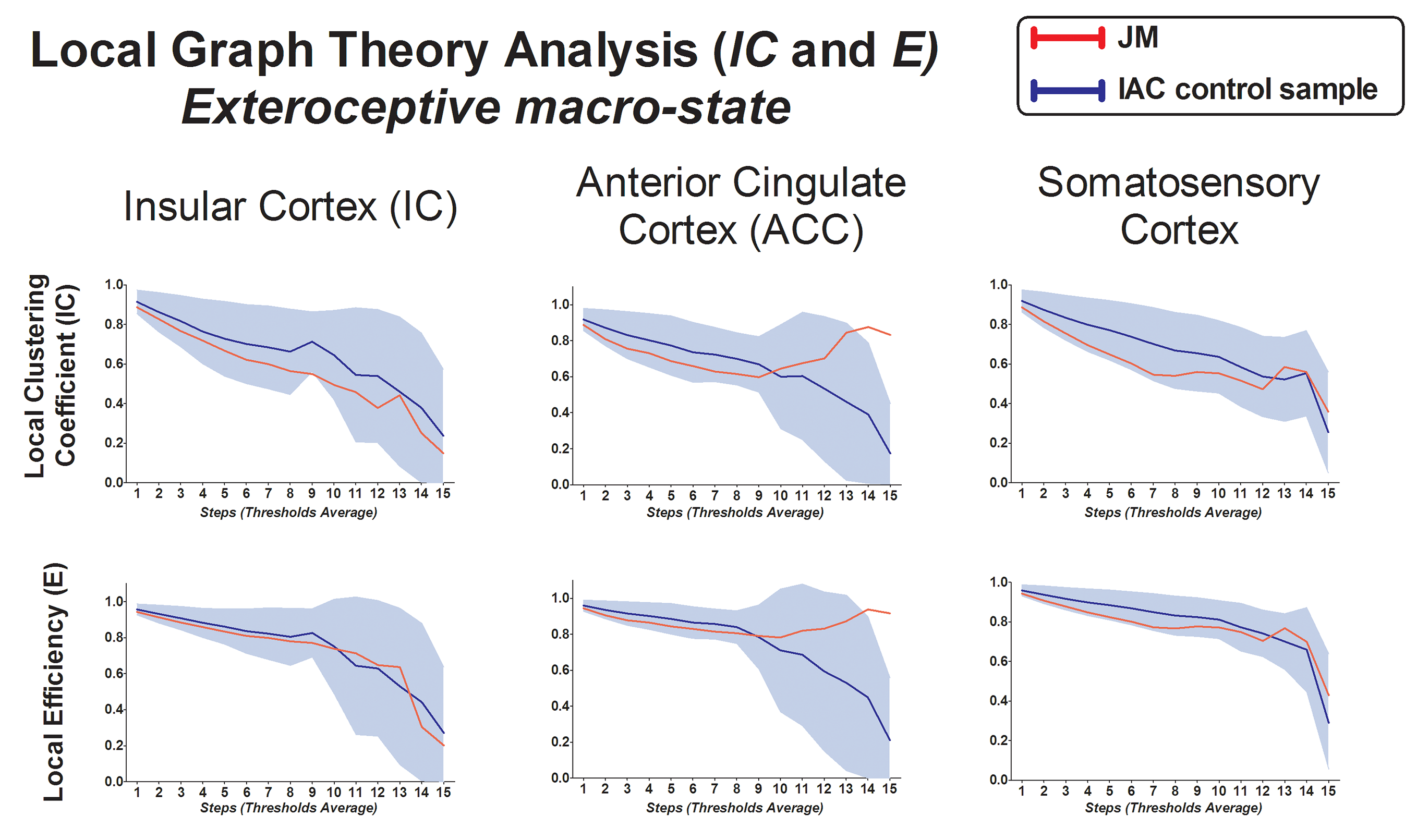

Supplement: Figure S2 — Local Graph Theory Analysis − Mind wandering macro-state. Columns indicate each ROI from the interoceptive-emotional network, and rows indicate each graph metric. The Y-axis shows raw metric scores, and the X-axis shows the range of thresholds, from 50 to 800, in steps of 50 (excluding extreme values where networks disaggregate). Boxes indicate significant and trend differences between JM and the control sample. Blue shadows represent controls' standard deviation area. (TIF) [file pone.0098769.s002.tif]

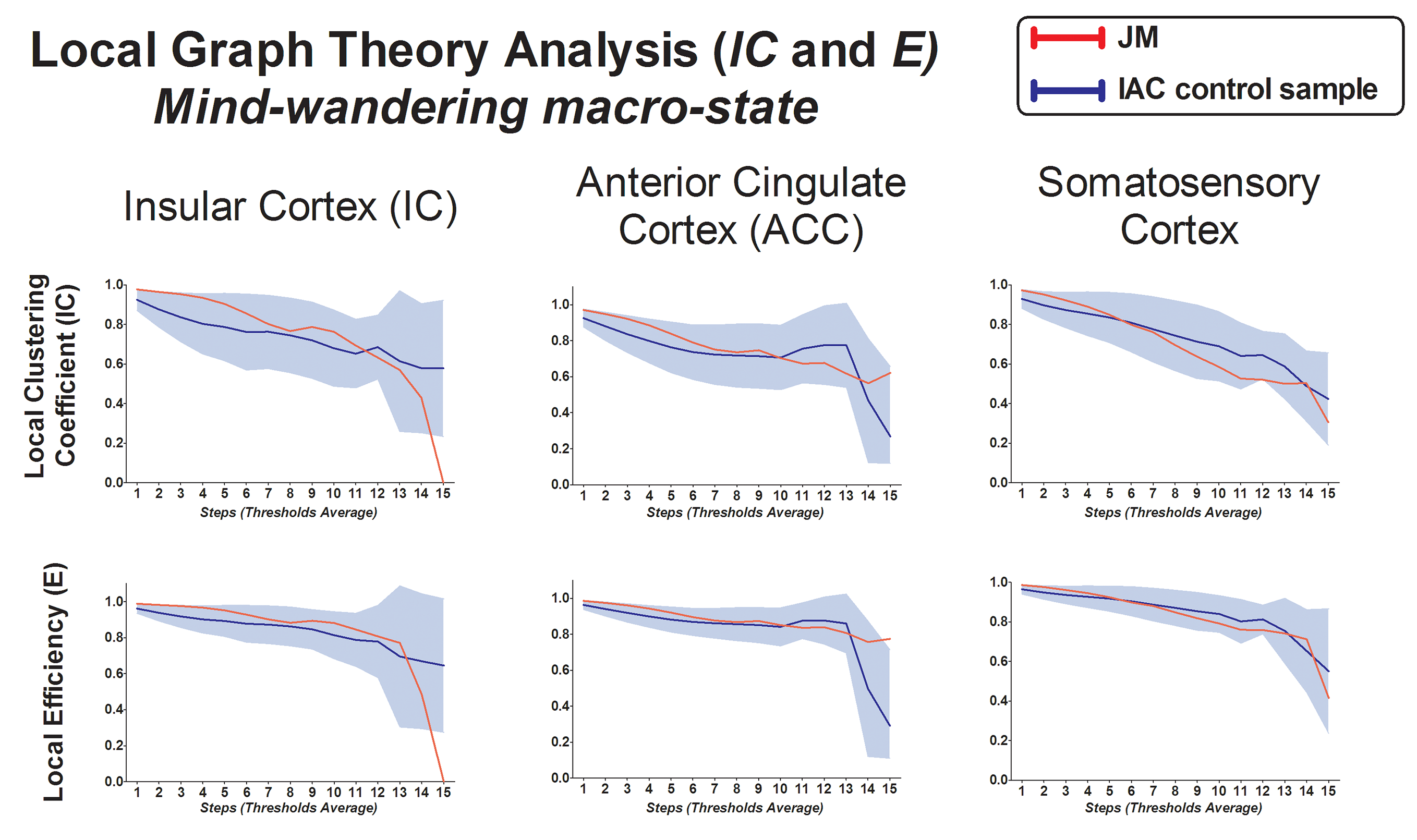

Supplement: Figure S3 — Local Graph Theory Analysis − Exteroceptive macro-state. Columns indicate each ROI from the interoceptive-emotional network, and rows indicate each graph metric. The Y-axis shows raw metric scores, and the X-axis shows the range of thresholds, from 50 to 800, in steps of 50 (excluding extreme values where networks disaggregate). Boxes indicate significant and trend differences between JM and the control sample. Blue shadows represent controls' standard deviation area. (TIF) [file pone.0098769.s003.tif]
