# Supplementary material for: How Do You Feel when You Can't Feel Your Body? Interoception, Functional Connectivity and Emotional Processing in Depersonalization-Derealization Disorder
Source: PLoS One. 2014 Jun 26;9(6):e98769. doi: 10.1371/journal.pone.0098769 (PMC4072534; doi:10.1371/journal.pone.0098769)
Supplement: Information S1 — 1000 Functional Connectomes Project's analysis and results. Description of the analysis and results of the comparison between the control sample and the data from the 1000 Functional Connectome Project. (DOC) [file pone.0098769.s004.doc]

**Information S1.**

To test if the five subjects from IAC group could be considered a representative control sample, we compared their large-scale functional organization against a data set of control subjects downloaded from the 1000 Functional Connectomes Project , an open-access repository of resting-state functional MRI datasets (<http://fcon_1000.projects.nitrc.org/>).

First Ann Arbor data set was chosen to run this comparison given its similarities in the fMRI acquisition (TR = 2000 ms, axial slices = 40 and time points = 295) and in demographic characteristics (n=23 −21 males /2 females−; mean age = 20.71; all right handed, except for one ambidextrous subject) with the control group used in this study.

Mind-wandering is the only cognitive macro state available in data sets from the connectomes project. Given this, both samples were analyzed and compared in this state. We applied the same procedures and methodologies detailed in Materials and method section for the functional images preprocessing, the creation of 116-node functional connectivity networks and graph theory analysis.

Repeated measure ANOVA was performed and Tukey’s HSD posthoc test for path length (L), clustering coefficient (C), degree (K), and small world (SW, same measure used in the case report), with group and thresholds (grouped together in steps of 50 thresholds, from 50 to 800) as independent factors.

Fig. suppl. 1 shows that no significant differences were found between groups in none of the FC metrics (L, F(14, 364) = 0.79, p = 0.67; C, F(14,364) = 0.67, p = 0.80; K, F(14,364) = 0.46, p = 0.95; SW, F(14,364) = 0.92, p = 0.54) and in none of the different thresholds selected. Post-hoc analyses evidenced a significant effect of thresholds (grouped in steps) for all metrics, however, as presented before, interaction between group and threshold was never significant. This suggests that threshold selection did not change the pattern observed across groups.

These FC metrics results support our assumption that subjects from the sample IAC present a similar large-scale functional organization that a larger group of healthy subjects. In conclusion, despite the small sample size, results suggest that the IAC control group might be representative of a more general healthy population.
