# Supplementary material for: How Do You Feel when You Can't Feel Your Body? Interoception, Functional Connectivity and Emotional Processing in Depersonalization-Derealization Disorder
Source: PLoS One. 2014 Jun 26;9(6):e98769. doi: 10.1371/journal.pone.0098769 (PMC4072534; doi:10.1371/journal.pone.0098769)
Supplement: Information S2 — Graph theory metrics: Local Networks of Interoceptive Condition. Detailed results of graph analysis of the IC, ACC and somatonsesory cortex during the interoceptive condition. (DOC) [file pone.0098769.s005.doc]

**Information S2.**

*Graph theory metrics: Local Networks of Interoceptive Condition*

In the local metric analysis of the ROIs from the interoceptive-emotional network, we only found trend and significant differences in the interoceptive macro-state (see Fig 6). In the IC, the patient presented a significant decreased of the local clustering coefficient (lC) and of the local efficiency (E) in the eighth and ninth steps (for lC: steps 8, t = -2.78, p = 0.05, Z_cc_ = -3.05; 9, t = -2.78, p = 0.05, Z_cc_ = -3.05 // for E: steps 8, t = -2.75, p = 0.05, Z_cc_ = -3.02; 9, t = -2.70, p = 0.05, Z_cc_ = -3.03). Regarding the ACC, we found in the patient lower values both on the local clustering coefficient (lC), only in the thirteenth step (13; t = -2.2, p = 0.09, Z_cc_ = -2.41), and in the local efficiency (E) (steps 11, t = -2.58, p = 0.06, Z_cc_ = -2.83; 12, t = -5.40, p < 0.01, Z_cc_ = -5.91; 13, t = -2.44, p = 0.09, Z_cc_ = -2.44) than controls. Finally, in the somatosensory cortex, JM presented diminished values respect the AIC sample in the last steps of both the local clustering coefficient (lC, steps 11, t = -2.15, p = 0.09, Z_cc_ = -2.36; 12, t = -2.98, p = 0.04, Z_cc_ = -3.26; 13, t = -2.37, p = 0.07, Z_cc_ = -2.59; 14 t = -5.11, p < 0.01, Z_cc_ = -5.60; 15, t = -5.59, p < 0.01 , Z_cc_ = -6.12) and the local efficiency (E, steps 11, t = -2.46, p = 0.07, Z_cc_ = -2.70; 12, t = -4.29, p = 0.01, Z_cc_ = -4.70; 13, t = -5.87, p < 0.01 , Z_cc_ = -6.43; 14, t = -19.43, p < 0.01, Z_cc_ = -21.28; 15, t = -18.17, p < 0.01, Z_cc_ = -20.50).
