# Supplementary material for: How Do You Feel when You Can't Feel Your Body? Interoception, Functional Connectivity and Emotional Processing in Depersonalization-Derealization Disorder
Source: PLoS One. 2014 Jun 26;9(6):e98769. doi: 10.1371/journal.pone.0098769 (PMC4072534; doi:10.1371/journal.pone.0098769)
Supplement: Information S3 — Empathy for Pain (EPT) Results. Table with the complete results of this task. (DOC) [file pone.0098769.s006.doc]

**Information S3**

Empathy for pain task. Ratings and reaction times.

|  |  | **JM** | **EAC Sample**  **(mean±SD)** | ***t; p; Zcc*** |
| --- | --- | --- | --- | --- |
| **Rating Neutral Situation** | Intentionality | 33.33* | 100 ± 1 | *t* = -60.87, *p* < 0.01, *Zcc* = -66.67 |
|  | Empathic Concern | -4.56* | -8.33 ± 1 | *t*= 3.44; *p*=0.01; *Zcc*= 3.77 |
|  | Discomfort | -7.22* | -8.31 ± 0.04 | *t* = 20.04; *p*< 0.01; *Zcc* = 22.24 |
|  | Harmful Behaviour | -8.33 | -8.33 ± 1 | *t* = 20.04; *p*= 0.5; *Zcc* = 0 |
|  | Valence Behaviour | -0.67* | 5.15 ± 1,95 | *t* = -2.72; *p*= 0.02; *Zcc* = -2.98 |
|  | Correctness | -5.22* | -8.33 ± 1 | *t* = 2.84; *p*= 0.02; *Zcc* = 3.11 |
|  | Punishment | -8.33 | -8.33 ± 1 | *t* = 0.00; *p*= 0.5; *Zcc* = 0 |
| **Rating Intentional**  **Situation** | Intentionality | 90.91 | 98.18 ± 4.06 | *t* = -1.63, *p* = 0.09, *Zcc* = -1.79 |
|  | Empathic Concern | -5.03* | 1.53 ± 1.43 | *t* = -4.18; *p*<0.01; *Zcc* = -4.59 |
|  | Discomfort | -5.64* | 1.51 ± 1.62 | *t* = -4.02; *p* <0.01; *Zcc* = -4.40 |
|  | Harmful Behaviour | 2.45 | 2.86 ± 1.64 | *t* = -0,23; *p*= 0.41; *Zcc* = -0.25 |
|  | Valence Behaviour | 0.97 | 2.42 ± 1.13 | *t* = -1.17; *p*= 0.15; *Zcc* = -1.28 |
|  | Correctness | 4.97 | 3.79 ± 0.91 | *t* = 1.16; *p*= 0.15; *Zcc* = -1.28 |
|  | Punishment | 2.76 | 2.08 ± 1.38 | *t* = 0.44; *p*= 0.33; *Zcc* = 0.49 |
|  | Intentionality | 100 | 92.72 ± 7.60 | *t* = 0.87, *p* = 0.21, *Zcc* = 0.95 |
| **Ratings Accidental Situations** | Empathic Concern | -3.09 | -3.13 ± 3.03 | *t* = 0.01; *p*= 0.49; *Zcc* = 0.13 |
|  | Discomfort | -4.12 | -4.12 ± 3.10 | *t* = 0.00; *p*= 0.49; *Zcc* = 0.003 |
|  | Harmful Behaviour | -7.12 | -5.99 ± 2.89 | *t* = -0.35; *p*=0,36; *Zcc* = -0.39 |
|  | Valence Behaviour | -7.91 | -5.18 ± 2.60 | *t* = -0.95; *p*= 0.19; *Zcc* = -1.05 |
|  | Correctness | 1.76 | -4.56 ± 3.41 | *t* = 1.69; *p*= 0.08; *Zcc* = 1.85 |
|  | Punishment | -8.3 | -5.57 ± 3.38 | *t* = -0.73; *p*= 0.25; *Zcc* = -0.80 |
|  | Intentionality | 4291.33 | 2992.73 ± 3247.74 | *t*= 0.36; *p*=0.36*; Zcc*=0.4 |
| **Reaction Time Neutral Situation** | Empathic Concern | 3520.33 | 3615.6 ± 1808.66 | *t* = 0.04; *p*= 0.48; *Zcc* = -0.05 |
|  | Discomfort | 1955.33 | 1483.13 ± 746.05 | *t* = 0.57; *p*= 0.29; *Zcc* = 0.63 |
|  | Harmful Behaviour | 2835.33* | 1744.93 ± 383 | *t* = 2.59; *p*= 0.03; *Zcc* = 2.84 |
|  | Valence Behaviour | 2282.67 | 2758.86 ± 1479.65 | *t* = -0.29; *p*= 0.39; *Zcc* = -0.32 |
|  | Correctness | 2697.67 | 2483.73 ± 1209.86 | *t* = 0.16; *p*= 0.43; *Zcc* = 0.17 |
|  | Punishment | 1549 | 1951.8 ± 605.12 | *t* = -0.60; *p*= 2.88; *Zcc* = -0.66 |
|  | Intentionality | 2604.64 | 1659.81 ± 543.46 | *t*= 1.58*; p=*0.09*; Zcc*= 1.73 |
| **Reaction Time Intentional** | Empathic concern | 3982.55 | 2851.94 ± 762.31 | *t* = 1.35; *p*= 0.12; *Zcc* = 1.48 |
|  | Discomfort | 2241.82 | 2355.50 ± 893.06 | *t* = -0.11; *p*= 0.45; *Zcc* = -0.12 |
|  | Harmful Behaviour | 2926.36 | 2337.16 ± 358.41 | *t* = 1.50; *p*= 0.10; *Zcc* = 1.64 |
|  | Valence Behaviour | 3064.82 | 4002.16 ± 1528.79 | *t* = -0.55; *p*= 0.30; *Zcc* = -0.61 |
|  | Correctness | 2522.55 | 2195.6 ± 764.65 | *t* = 0.39; *p*= 0.35; *Zcc* = 0.42 |
|  | Punishment | 2874 | 8401.36 ± 12789.2 | *t* = -0.39; *p*= 0.41; *Zcc* = 0.24 |
|  | Intentionality | 1928.55 | 2753.92 ± 1409.49 | *t*= -0.53*; p*= 0.31*; Zcc*= -0.58 |
| **Reaction Time Accidental** | Empathic Concern | 2650.45 | 2791.09 ± 1293.62 | *t* = -0.09; *p*= 0.46; *Zcc* = -0.10 |
|  | Discomfort | 1756.27 | 1953.41 ± 1054.19 | *t* = -0.17; *p*= 0.43; *Zcc* = -0.18 |
|  | Harmful Behaviour | 2774.55 | 1653.94 ± 911.78 | *t* = 1.12; *p*= 0.16; *Zcc* = 1.22 |
|  | Valence Behaviour | 2949.73 | 2628.29 ± 1137.67 | *t* = 0.25; *p*= 0.40; *Zcc* = 0.28 |
|  | Correctness | 2382.18 | 2206.45 ± 792.34 | *t* = 0.20; *p*= 0.42; *Zcc* = 0.22 |
|  | Punishment | 2194 | 1734.16 ± 626.94 | *t* = 0.66; *p*= 0.26; *Zcc* = 0.73 |

*RT in miliseconds*

** significant results.*
